# Anatomical distribution and severity of dental caries by tooth and surface in preschool children from Costa Rica: a cross-sectional descriptive study

**DOI:** 10.1186/s12903-025-06906-2

**Published:** 2025-09-29

**Authors:** Adrian Gomez-Fernandez

**Affiliations:** Faculty of Dentistry, University of Costa Rica, San José, Costa Rica

**Keywords:** Dental caries, Primary teeth, Tooth surfaces, Caries severity, ICDAS, Preschool children, Costa Rica, Risk-based prevention

## Abstract

**Background:**

Understanding the anatomical distribution and severity of dental caries is crucial for developing targeted preventive and therapeutic strategies in pediatric populations. While global prevalence studies offer a general overview of disease burden, few have explored caries patterns by tooth type and surface in young children. This study aimed to assess the distribution and severity of dental caries lesions in primary teeth among Costa Rican preschool children enrolled in public early childhood centers.

**Methods:**

A cross-sectional descriptive study was conducted through a secondary analysis of data from an oral health survey involving 803 children under 81 months of age attending government-run early childhood care and nutrition centers, which serve low-income populations in Costa Rica. Calibrated dentists conducted clinical examinations using the International Caries Detection and Assessment System (ICDAS). Each tooth was evaluated individually, with the most severely affected surface recorded for analysis. Lesions were classified as noncavitated (ICDAS codes 1–2) or cavitated (codes 3–6). Descriptive statistics were performed to analyze the frequency and severity of carious lesions by tooth and surface.

**Results:**

The second primary molars were the most frequently affected teeth, accounting for 15.81% (95% confidence interval (CI): 13.4–18.2) of all caries-affected surfaces, followed by the first primary molars (11.56%). The highest average severity was observed in the upper central incisors (mean ICDAS = 4.25, 95% CI: 4.10–4.40). Occlusal surfaces were the most commonly affected (18.09%), whereas buccal surfaces represented the largest proportion of total lesions (39.32%). Although distal surfaces were less frequently affected (2.38%), they exhibited the highest average severity (4.86). Noncavitated caries lesions were predominant, especially on the second molars and buccal surfaces, while proximal surfaces had a greater proportion of advanced lesions.

**Conclusions:**

Second primary molars were the most frequently affected teeth, while upper central incisors exhibited the highest caries lesion severity. Occlusal and buccal surfaces together accounted for the largest proportion of carious lesions. These findings underscore the need for preventive strategies tailored to anatomical risk patterns in preschool populations.

## Introduction

Identifying the anatomical location of dental caries in the primary dentition is a fundamental step in optimizing prevention, early diagnosis, and treatment planning. Although general prevalence studies offer a broad overview of disease burden, detailed analyses of individual teeth and surfaces reveal specific patterns in caries distribution, thereby informing both clinical decision-making and public health strategies [1, 2]. Classic studies have demonstrated that the location and distribution of lesions, particularly in primary teeth, are not random, but instead reflect underlying etiological and behavioral factors [3, 4]. Therefore, evaluating caries at the tooth and surface level is essential for identifying high-risk anatomical sites and implementing risk-based, targeted interventions.

Dental caries is a multifactorial and transmissible disease with a high prevalence in childhood and is regarded as a major global public health concern due to its short- and long-term consequences [5, 6]. In addition to damaging dental structures, caries adversely affects children's quality of life by impairing nutrition, sleep, growth, and academic performance [7, 8]. While traditional surveillance typically focuses on overall prevalence, analyses of specific tooth types and surfaces enable the identification of high-risk sites and the prioritization of cost-effective interventions.

International studies have consistently reported that primary molars are the most frequently affected teeth, especially on their occlusal and proximal surfaces, due to their retentive morphology and the difficulty of maintaining proper oral hygiene in these areas [9, 10]. Likewise, upper incisors have been associated with early childhood caries, which are frequently related to inappropriate dietary practices such as prolonged bottle feeding [11, 12]. Recent studies have also reported early involvement of the first permanent molars, emphasizing the need for preventive interventions during the early phases of mixed dentition [13]. In addition, enamel developmental defects, such as molar incisor hypomineralization (MIH), have been identified as factors that increase the vulnerability of these teeth and complicate their clinical management [14].

The International Caries Detection and Assessment System (ICDAS) has been validated as a sensitive and reliable tool for detecting and classifying caries lesions, ranging from early demineralization to advanced cavitation [15]. This system not only improves clinical documentation but also enables a more detailed assessment of lesion severity, an essential component of risk-based and minimally invasive approaches. The anatomical distribution of caries has long been a topic of interest; early studies of permanent dentition in older children identified specific vulnerability patterns in incisor teeth [3, 16]. However, these early studies lacked the diagnostic precision of modern systems such as the ICDAS, which now allows for more accurate and clinically meaningful assessments of primary dentition.

In Costa Rica, recent analyses indicate that dental caries continue to be the most prevalent oral disease among children, particularly in populations with limited access to preventive care [17]. Despite national efforts to improve oral health indicators, disparities remain, particularly among preschool-aged children from low-income backgrounds. A study carried out in two school communities in the Poas region revealed that 27% of children had active caries in teeth 4.6, and prevalence increased with age. Furthermore, a statistically significant association was observed between father’s education level and the children’s oral health status [18]. Additional evidence of this disparity was reported in a study of children and adolescents living in foster care institutions in San José, where the prevalence of caries reached 96.35%, with early enamel lesions being the most frequently observed finding [19]. Although national data show modest improvements over time, social vulnerability remains a critical determinant of disease burden. Education and Nutrition Centers and Comprehensive Child Care Centers (Centros de Educación y Nutrición y Centros Infantiles de Atención Integral – CEN-CINAI), which serve children in socially at-risk environments, continue to represent priority settings for the implementation of effective and sustainable preventive strategies grounded in local evidence. This scenario is consistent with findings from other Latin American and Caribbean countries, where socioeconomic factors significantly influence both the distribution of caries and the effectiveness of community-based interventions [20].

This type of analysis aligns with the principles of risk-based dentistry, in which identifying specific anatomical patterns enables the anticipation of disease progression and the establishment of personalized care models [21, 22]. This approach promotes the implementation of targeted preventive and therapeutic strategies that focus on the most susceptible teeth and surfaces, thereby optimizing clinical and public health resources. Recent studies on early childhood caries also emphasize the importance of assessing individual behavioral and environmental risk factors to guide clinical decision-making in young children [23].

Despite the clinical value of the ICDAS system in detecting early and advanced lesions, few studies have used this approach to systematically assess dental caries distribution and severity by tooth surface in young populations. Recent research from Nepal, Greece, and Colombia has demonstrated the usefulness of ICDAS in mapping lesion patterns by surface in different age groups and settings [24–26]. However, data from Latin American preschoolers remain limited.

Therefore, the objective of this study was to analyze the anatomical distribution and severity of dental caries in the primary dentition of Costa Rican preschoolers using the ICDAS system, classifying lesions by affected tooth and surface, in order to complement general prevalence data with clinically relevant information for risk-based prevention and early intervention strategies.

## Materials and methods

### Study design and population

This cross-sectional descriptive study is based on a secondary analysis of data collected during an oral health survey conducted from March to June 2013 among children enrolled in the national network of Education and Nutrition Centers and Comprehensive Child Care Centers (CEN-CINAI, by its Spanish acronym). These centers serve socioeconomically disadvantaged families across all regions of Costa Rica, providing a strategic platform for assessing oral health conditions in this vulnerable population.

A two-stage stratified cluster sampling method was employed. In the first stage, 40 centers were selected from a national registry of 390 eligible CEN-CINAI establishments using probability proportional to size (PPS), with stratification by national planning region (Central, Chorotega, Central Pacific, Brunca, Huetar Atlantic, and Huetar North). A disproportionate allocation strategy was applied, assigning 45% of the sample to the Central region and distributing the remaining 55% proportionally among the other five regions.

In the second stage, a systematic subsample of up to 25 children was selected within each center using a circular sampling method. If fewer than 25 children were present on the day of examination, all were included. This two-stage design ensured that all children under 81 months had an equal probability of selection.

**Fig. 1:**
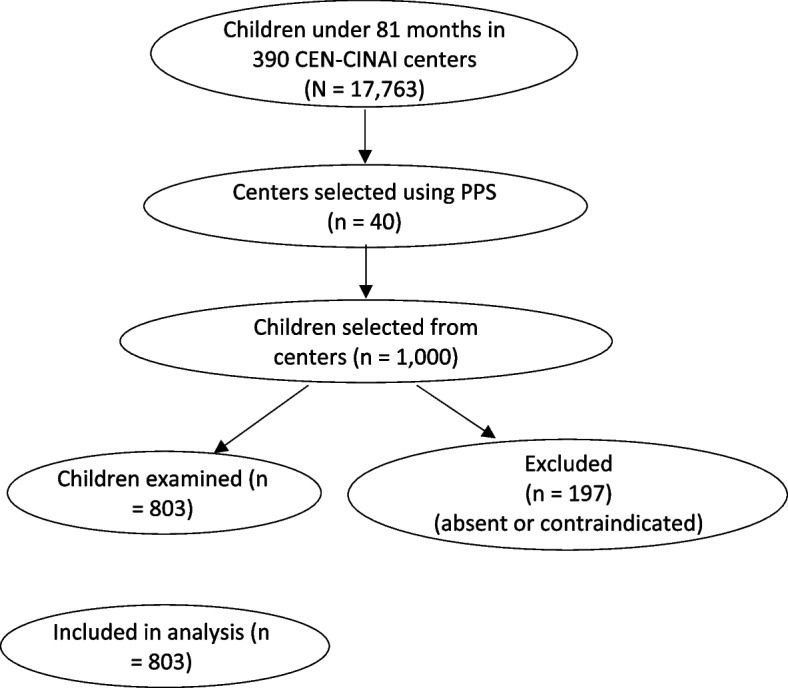
Flow diagram of participant selection and inclusion. The study used a two-stage probabilistic cluster sampling method based on the national registry of children enrolled in CEN-CINAI centers. From a total of 17,763 children under 81 months across 390 centers, 40 centers were selected. Within these, 1,000 children were invited to participate. A total of 803 children were clinically examined and included in the final analysis. Children were excluded if absent on the day of the exam or presented medical contraindications

Although the original sampling plan aimed to include 1,000 children enrolled in CEN-CINAI centers across Costa Rica, logistical and operational limitations resulted in a final sample of 803 children. A post hoc estimation indicated that this sample size was sufficient to estimate a dental caries prevalence of 80% with a 3% margin of error and 95% confidence level, assuming simple random sampling within the target population. Figure 1 summarizes the selection and inclusion process.

### Data collection and inclusion criteria

Clinical examinations were performed on-site at selected CEN-CINAI centers using portable dental units under standardized field conditions. Each clinical team comprised a calibrated dentist who served as the examiner, a recorder, and an assistant responsible for pre-examination oral hygiene using fluoride-free toothpaste. Lighting was provided by headlamps, and instruments included a #5 plain dental mirror and a WHO 11.5 probe, used only when necessary to avoid enamel damage. A dedicated sterilized instrument set was used for each patient during the session, and all instruments were reprocessed according to national infection control protocols at the end of each clinical day.

Eight general and pediatric dentists conducted the examinations. All were previously trained and calibrated in the use of the ICDAS system by internationally certified gold standard trainers. A calibration pilot with 30 children (not included in the final sample) was conducted, yielding inter- and intra-examiner kappa coefficients ranging from 0.80 to 0.93. Periodic reinforcement sessions were held during the study to maintain diagnostic consistency.

This study focused exclusively on the primary dentition. All present primary teeth were examined; permanent teeth, if present, were excluded. Clinical data were recorded using pre-coded forms specifically designed for this study.

### Classification by Tooth type and tooth surface

Each primary tooth was evaluated individually, without grouping by anatomical category (incisor, canine, or molar), in order to identify specific patterns in lesion distribution. The analyzed surfaces included the occlusal, mesial, distal, buccal/vestibular, and palatal/lingual areas. For surface-level analysis, each surface was assessed independently. For tooth-level analysis, only the surface with the highest ICDAS score per tooth was retained to avoid duplication of lesions.

### Caries detection and severity scoring

Caries lesions were diagnosed using the International Caries Detection and Assessment System (ICDAS). Each surface—including occlusal, mesial, distal, buccal/vestibular, palatal/lingual, buccal pit, and palatal groove—was examined individually.

Lesions were classified as follows:

- ICDAS codes 1–2: noncavitated (incipient) lesions
- ICDAS codes 3–6: cavitated lesions, with increasing levels of severity

### Severity score calculation

ICDAS scores were treated as ordinal variables ranging from 1 (initial visual enamel change) to 6 (extensive cavitation with visible dentin). For the purpose of severity analysis:

- At the tooth level, only the most severe score per tooth was used.
- At the surface level, all surfaces were analyzed independently.

Lesions were also grouped into two clinical categories: noncavitated (ICDAS 1–2) and cavitated (ICDAS 3–6), representing early and advanced stages of disease, respectively.

### Bias control

Several measures were implemented to minimize potential sources of bias. The two-stage sampling design based on probability proportional to size (PPS) ensured geographic and demographic representation. Within each center, systematic sampling reduced selection bias. Standardized clinical protocols were applied uniformly across all sites, including examiner positioning, lighting conditions, examination instruments, and data collection procedures. Diagnostic reliability was maintained through initial and ongoing examiner calibration. Data entry was subject to random verification and cross-checking against original clinical forms to detect and resolve inconsistencies.

### Variables and data structure

The dataset included tooth-level variables, specifically tooth identifier, surface designation, and corresponding ICDAS score. No sociodemographic variables at the child level were available, as the analysis was based on secondary data originally collected for clinical surveillance purposes.

### Statistical analysis

Descriptive statistics were used to calculate the absolute and relative frequencies of caries lesions by tooth and surface. All analyses were conducted using SPSS software, version 28.0 (IBM Corp., Armonk, NY, USA).

Data entry was performed by a trained team using standardized pre-coded forms. Random verification procedures were implemented to ensure data accuracy, and any inconsistencies were resolved through review of the original clinical records.

### Ethical considerations

The original study was approved by the Scientific Ethics Committee of the University of Costa Rica (approval number VI-3058–2012). Written informed consent was obtained from the parents or legal guardians of all participants prior to examination. Data confidentiality was ensured through anonymization prior to analysis. This study complied with the ethical principles set forth in the Declaration of Helsinki for research involving human participants. Clinical trial number: not applicable.

## Results

The detailed distribution of dental caries in primary teeth, analyzed by individual tooth and lesion severity, highlights important differences in both frequency and severity. Among all primary tooth types, maxillary molars, both right and left, had the highest number of caries-affected surfaces (Table 1).

**Table 1:** Distribution and severity of dental caries in primary teeth according to individual tooth and ICDAS code

Tooth	Caries Code 1	Caries Code 2	Caries Code 3	Caries Code 4	Caries Code 5	Caries Code 6	Total Affected	Percentage of Affected (%)	Average Severity
Upper right second molar	51 (8.9%)	332 (57.9%)	41 (7.2%)	44 (7.7%)	58 (10.1%)	47 (8.2%)	573	11.89	2.77
Upper right first molar	16 (4.2%)	169 (44.4%)	18 (4.7%)	20 (5.2%)	65 (17.1%)	93 (24.4%)	381	9.49	3.6
Upper right canine	10 (5.1%)	144 (73.5%)	18 (9.2%)	3 (1.5%)	13 (6.6%)	8 (4.1%)	196	6.1	2.43
Upper right lateral incisor	9 (2.8%)	148 (45.3%)	26 (8.0%)	14 (4.3%)	52 (15.9%)	78 (23.9%)	327	10.18	3.57
Upper right central incisor	13 (2.8%)	142 (30.7%)	29 (6.3%)	30 (6.5%)	140 (30.2%)	109 (23.5%)	463	14.41	4.01
Upper left central incisor	9 (1.9%)	141 (29.7%)	40 (8.4%)	34 (7.2%)	134 (28.2%)	117 (24.6%)	475	14.79	4.04
Upper left lateral incisor	15 (4.1%)	154 (42.3%)	20 (5.5%)	8 (2.2%)	71 (19.5%)	96 (26.4%)	364	11.33	3.7
Upper left canine	3 (1.7%)	126 (70.4%)	19 (10.6%)	3 (1.7%)	17 (9.5%)	11 (6.1%)	179	5.57	2.65
Upper left first molar	26 (6.4%)	156 (38.6%)	17 (4.2%)	31 (7.7%)	66 (16.3%)	108 (26.7%)	404	10.06	3.69
Upper left second molar	50 (8.8%)	368 (65.1%)	31 (5.5%)	37 (6.5%)	31 (5.5%)	48 (8.5%)	565	11.73	2.6
Lower right second molar	46 (5.4%)	627 (73.2%)	42 (4.9%)	27 (3.2%)	57 (6.7%)	58 (6.8%)	857	17.79	2.53
Lower right first molar	28 (5.4%)	265 (50.8%)	21 (4.0%)	27 (5.2%)	93 (17.8%)	88 (16.9%)	522	13	3.3
Lower right canine	5 (4.8%)	81 (77.1%)	5 (4.8%)	1 (1.0%)	7 (6.7%)	6 (5.7%)	105	3.27	2.45
Lower right lateral incisor	2 (2.6%)	53 (69.7%)	5 (6.6%)	4 (5.3%)	8 (10.5%)	4 (5.3%)	76	2.37	2.67
Lower right central incisor	1 (1.3%)	37 (47.4%)	7 (9.0%)	12 (15.4%)	16 (20.5%)	5 (6.4%)	78	2.43	3.26
Lower left central incisor	1 (1.2%)	43 (51.2%)	7 (8.3%)	10 (11.9%)	19 (22.6%)	4 (4.8%)	84	2.62	3.18
Lower left lateral incisor	4 (6.0%)	48 (71.6%)	1 (1.5%)	3 (4.5%)	7 (10.4%)	4 (6.0%)	67	2.09	2.6
Lower left canine	7 (5.9%)	80 (67.2%)	10 (8.4%)	4 (3.4%)	12 (10.1%)	6 (5.0%)	119	3.7	2.6
Lower left first molar	15 (3.0%)	254 (51.5%)	19 (3.9%)	30 (6.1%)	83 (16.8%)	92 (18.7%)	493	12.28	3.38
Lower left second molar	46 (5.5%)	605 (72.2%)	46 (5.5%)	26 (3.1%)	66 (7.9%)	49 (5.8%)	838	17.39	2.53

Distribution of caries lesions by ICDAS severity code for each primary tooth. Each cell shows the absolute number of surfaces with a given code, followed by the percentage of affected surfaces for that code in parentheses. The last three columns show the total number of affected surfaces per tooth, the percentage of affected surfaces relative to all evaluated surfaces, and the mean severity score

For instance, the upper right second primary molar accounted for 573 affected surfaces (11.89% of all evaluated surfaces), with an average severity score of 2.77. Similarly, the upper right first primary molar exhibited 381 affected surfaces (9.49%). Among anterior teeth, the maxillary central and lateral incisors had slightly lower frequencies but higher severity scores, reaching up to 4.01.

The upper right and left central incisors had the highest average severity scores (4.01 and 4.04, respectively), indicating greater lesion severity in anterior teeth than in primary molars.

When grouped by type, second primary molars had the highest proportion of affected surfaces (12.9%), followed by first primary molars (11.4%), while canines had the lowest percentage (6.1%) (Table 2).

**Table 2:** Distribution of dental caries in primary teeth by tooth group and ICDAS Code

Group	Caries Code 1	Caries Code 2	Caries Code 3	Caries Code 4	Caries Code 5	Caries Code 6	Total Affected	Percentage of Affected (%)	Average Severity
Central incisor	24 (2.2%)	363 (33.0%)	83 (7.5%)	86 (7.8%)	309 (28.1%)	235 (21.4%)	1100	8.783136	3.907273
Lateral incisor	30 (3.6%)	403 (48.3%)	52 (6.2%)	29 (3.5%)	138 (16.5%)	182 (21.8%)	834	6.686979	3.465228
Canine	25 (4.2%)	431 (72.0%)	52 (8.7%)	11 (1.8%)	49 (8.2%)	31 (5.2%)	599	4.801218	2.534224
First primary molar	85 (4.7%)	844 (46.9%)	75 (4.2%)	108 (6.0%)	307 (17.1%)	381 (21.2%)	1800	11.56441	3.472778
Second primary molar	193 (6.8%)	1932 (68.2%)	160 (5.6%)	134 (4.7%)	212 (7.5%)	202 (7.1%)	2833	15.80739	2.592658

Each cell represents the number of tooth surfaces affected by each ICDAS code, and the corresponding percentage of affected surfaces is shown in parentheses. The final columns show the total number of affected surfaces per tooth group, the percentage of affected surfaces relative to all evaluated surfaces, and the average ICDAS severity score for each group

In terms of lesion severity, the first primary molars had an average score of 3.12, while the second primary molars had an average of 2.98. Although maxillary molars were the most frequently affected, mandibular second molars also exhibited high frequencies, with 17.79% on the right and 17.39% on the left, and similar severity scores.

At the surface level, the occlusal surface was the most frequently affected (18.09%), followed by the buccal, mesial, distal, and palatal/lingual surfaces.

A detailed analysis of ICDAS severity codes revealed that in the upper right second molar, noncavitated lesions visible when wet (code 2) accounted for 57.9%, while cavitated lesions with visible dentin (code 5) represented 10.1%. In the upper right first molar, 44.4% of the lesions were code 2, whereas 24.4% were advanced lesions (code 6). This distribution indicated that most lesions were in early or moderate stages, although a notable proportion of advanced lesions with extensive dentin involvement was also observed.

**Fig. 2:**
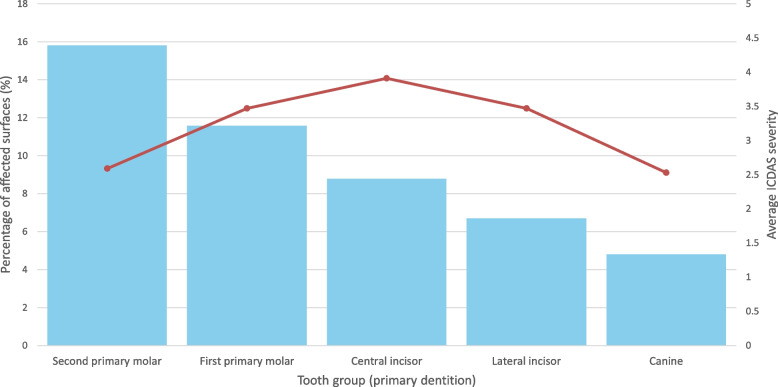
Percentage of affected surfaces and average ICDAS severity by tooth group in the primary dentition Bar chart depicting the percentage of affected tooth surfaces by group (left Y-axis) and the corresponding average ICDAS severity scores (right Y-axis) in Costa Rican preschool children. The second primary molars had the highest percentage of affected surfaces, whereas the central incisors had the greatest mean severity. This combined visualization highlights both the distribution and severity of dental caries across different primary tooth groups

Analysis by tooth group revealed that second primary molars had the highest percentage of affected surfaces (15.81%), followed by first primary molars (11.56%) and central incisors. Canines exhibited the lowest proportion (4.80%). This pattern is illustrated in Fig. 2, which also displays the corresponding average ICDAS severity scores for each tooth group. The detailed numerical values are provided in Table 2.

In terms of average severity, central incisors had the highest score (3.91), followed by lateral incisors and first primary molars, each with a score of 3.47. Although the second primary molars were highly affected, they presented a lower average severity score (2.59).

In the second primary molars, 68.2% of the lesions were classified as code 2, and 5.6% were classified as code 3. The first primary molars exhibited 46.9% of the lesions in code 2 and 6.0% in code 4. Among central incisors, 33.0% had lesions in code 2, and 7.8% had lesions in code 4, whereas among lateral incisors, 48.3% had lesions in code 2, and 3.5% had lesions in code 4. Canines showed the highest proportion of initial lesions, with 72.0% in code 2 and 8.7% in code 3.

The analysis of caries on the tooth surface revealed anatomical differences in both lesion frequency and severity (Table 3). Occlusal surfaces exhibited the highest percentage of affected areas (18.09%), with an average severity score of 3.16. The buccal surface area was 13.04% of the total area, although the average severity was lower (2.49).

**Table 3:** Distribution and severity of dental caries by tooth surface in the primary dentition

Surface	Caries Code 1	Caries Code 2	Caries Code 3	Caries Code 4	Caries Code 5	Caries Code 6	Total Affected	Percentage of Affected (%)	Average Severity
Occlusal	157 (9.0%)	764 (43.8%)	168 (9.6%)	161 (9.2%)	276 (15.8%)	217 (12.4%)	1743	18.09	3.16
Mesial	31 (3.5%)	317 (36.2%)	61 (7.0%)	39 (4.5%)	175 (20.0%)	252 (28.8%)	875	4.54	3.88
Distal	4 (0.9%)	75 (16.4%)	19 (4.1%)	19 (4.1%)	108 (23.6%)	233 (50.9%)	458	2.38	4.86
Buccal	122 (4.2%)	2237 (76.3%)	126 (4.3%)	69 (2.4%)	222 (7.6%)	157 (5.4%)	2933	13.04	2.49
Palatal or lingual	45 (3.8%)	601 (50.5%)	52 (4.4%)	82 (6.9%)	236 (19.8%)	174 (14.6%)	1190	5.29	3.32
*Palatal groove*	18 (18.0%)	56 (56.0%)	15 (15.0%)	3 (3.0%)	3 (3.0%)	5 (5.0%)	100	3.11	2.32
*Buccal pit*	20 (12.4%)	123 (76.4%)	9 (5.6%)	3 (1.9%)	2 (1.2%)	4 (2.5%)	161	5.01	2.11

Each cell shows the absolute number of surfaces affected by the ICDAS code, with the corresponding percentage in parentheses. The final columns indicate the total number of affected surfaces for each anatomical surface, the percentage of all affected surfaces represented by each surface type, and the average ICDAS severity score

Palatal and mesial surfaces had lower frequencies (5.29% and 4.54%, respectively), but higher severity scores (3.32 and 3.88, respectively). Distal surfaces, although the least frequently affected (2.38%), showed the highest average severity (4.86). The buccal pit and palatal groove surfaces accounted for smaller proportions of the total lesions (5.01% and 3.11%, respectively), with a predominance of incipient lesions.

Occlusal surfaces accounted for 23.36% of all carious surfaces (Table 4). Buccal surfaces, although affecting 13.04% of examined areas, contributed 39.32% of the total lesion burden. Palatal surfaces contributed 15.95% to the overall lesion burden.

**Table 4:** Contribution of affected tooth surfaces to total dental caries burden in the primary dentition

Surface	Total Affected Surfaces	Total Surfaces Evaluated	Percentage of Affected (%)	Contribution to Total Affected (%)
Buccal	2933	22,484	13.04	39.31635
Occlusal	1743	9636	18.09	23.36461
Palatal or lingual	1190	22,484	5.29	15.95174
Mesial	875	19,272	4.54	11.72922
Distal	458	19,272	2.38	6.13941
Palatal groove	161	3212	5.01	2.158177
Buccal pit	100	3212	3.11	1.340483

The buccal pit and mesial surfaces had lower frequencies (5.01% and 4.54%, respectively), although the mesial surfaces accounted for 11.73% of the total lesions. Although distal surfaces were the least affected, they played a significant role in severe caries cases.

## Discussion

This study identified distinct anatomical patterns in the prevalence and severity of dental caries among Costa Rican preschoolers. Second primary molars were the most frequently affected, while maxillary incisors exhibited the highest severity scores. Occlusal and buccal surfaces showed the highest lesion frequency, whereas distal surfaces had the greatest mean severity. These findings provide a clear basis for surface-specific preventive interventions.

The anatomical profile observed in this population highlights significant disparities in caries distribution and severity across tooth types and surfaces. The second primary molars were the most frequently affected teeth, while maxillary anterior teeth exhibited the highest severity scores—findings that reflect both the high prevalence and advanced stage of caries lesions in this vulnerable population. This pattern is consistent with the multifactorial and dynamic nature of dental caries, in which lesion progression is influenced by biological, behavioral, and social factors [27]. Moreover, the observed anatomical patterns align with international studies indicating that primary molars are especially susceptible due to their retentive morphology, posterior location, and challenges in maintaining oral hygiene [10, 20]. Similar trends have been reported in Indian preschoolers, where second primary molars had the highest dmfs (decayed, missing, and filled surfaces) scores in hierarchical pattern analyses [28].

Similar to the findings in other Latin American populations, where high caries rates in primary molars have been reported [13], the sample from these centers reflects a comparable pattern, particularly in second molars. A recent study of Mexican preschool and school-aged children also reported a high burden of caries—both in terms of prevalence and severity—based on conventional diagnostic criteria [29]. Additionally, a study conducted among Mexican schoolchildren using the ICDAS system confirmed the high prevalence and severity of lesions in second primary molars, reinforcing their priority status for preventive care [30]. A comparable trend was observed in West African pediatric populations, where mandibular molars, particularly the lower right first and second primary molars, showed the highest susceptibility to caries [31]. This pattern may be linked to socioeconomic vulnerability, as caries incidence has consistently been associated with lower income levels and limited access to preventive care [20]. Likewise, Colombian preschool children exhibited parallel anatomical patterns and severity scores, with maxillary molars and incisors being the most frequently affected. Surface-level ICDAS data from the same setting confirmed that occlusal surfaces of primary molars had the highest caries experience, significantly above other surfaces (*p* < 0.001) [32]. In a national survey conducted in Greece using ICDAS, schoolchildren (including those with primary teeth) also showed high frequencies of lesions on occlusal and proximal surfaces of mandibular molars, further supporting the surface-specific vulnerability of these sites [25]. These international findings mirror the anatomical patterns observed in Costa Rican children, reinforcing the value of surface-level mapping for targeted prevention.

In terms of severity, the maxillary central incisors had the highest mean ICDAS scores in this study, exceeding 4.0, with the upper left central incisor reaching 4.04. These findings align with previous studies on early childhood caries (ECC), in which maxillary anterior teeth are typically the first to be severely affected due to prolonged exposure to sugary liquids and inadequate oral hygiene practices [8, 12]. ECC has been strongly associated with microbial colonization by Streptococcus mutans and feeding behaviors during infancy, which exacerbate the progression of lesions in anterior teeth [12]. Similar results were observed in Myanmar preschoolers, where tooth-level analysis revealed high rates of decay in maxillary central incisors [33]. These findings underscore the importance of early and targeted preventive interventions that address both dietary habits and oral hygiene behaviors.

Regarding lesion distribution, the occlusal surface was the most frequently affected (18.09% of all evaluated surfaces), followed by the buccal (13.04%) and mesial (4.54%) surfaces. These results are consistent with previous evidence showing that occlusal surfaces are particularly vulnerable to caries due to their morphology and capacity for plaque retention [15, 22]. Moreover, metagenomic analyses have demonstrated that carious pit and fissure sites harbor distinct microbial communities enriched with cariogenic bacteria, reinforcing their role as ecological niches susceptible to dysbiosis in the absence of preventive measures [34]. The high proportion of advanced lesions (ICDAS codes 4–6) found on proximal surfaces reinforces existing evidence highlighting the difficulty of detecting interproximal caries in early stages without the use of adjunctive diagnostic tools [35]. Such anatomical patterns reinforce the need to focus preventive strategies on high-risk sites, particularly occlusal and buccal surfaces in primary molars.

A particularly noteworthy finding was the distal surface, which had the lowest frequency of caries (2.38%) but exhibited the highest mean severity (4.86). This pattern suggests that distal lesions are often diagnosed only at more advanced stages, likely due to their limited visibility during routine clinical examinations [15, 36].

Among less frequently affected anatomical sites, such as the buccal pit and palatal groove, this study revealed a predominance of incipient lesions. For instance, 76.4% of buccal pit lesions and 56.0% of palatal groove lesions were classified as ICDAS code 2. This observation aligns with findings of Featherstone [21], who noted that secondary anatomical features may serve as early sites of caries initiation when oral hygiene is inadequate.

These anatomical patterns have direct clinical implications for surface-based prevention strategies. The predominance of lesions on occlusal and buccal surfaces in molars highlights the importance of implementing pit and fissure sealants and promoting effective toothbrushing techniques targeting vestibular areas. The high severity of lesions on distal and mesial surfaces—despite their lower frequency—emphasizes the need for early interproximal caries detection, including the use of adjunctive diagnostic tools when appropriate. Educational interventions should focus on improving parents’ and caregivers’ awareness of high-risk surfaces and their early signs. In public health terms, preventive programs in CEN-CINAI centers should prioritize sealant application and supervised brushing protocols that consider surface-specific vulnerability, complementing general strategies based on common risk factors.

From a clinical perspective, these findings highlight the importance of administering pit and fissure sealants to high-risk primary molars, in accordance with guidelines from the American Academy of Pediatric Dentistry [8]. These results further support the implementation of risk-based prevention strategies that incorporate anatomical assessments to more effectively target the most vulnerable teeth and surfaces [10].

Compared to oral health programs in other countries that primarily focus on permanent dentition [37], this study underscores the need to prioritize primary molars in prevention and treatment strategies for Costa Rican children, especially those from socioeconomically disadvantaged backgrounds attending public early childhood centers. These data may help inform policymakers in allocating resources more efficiently and tailoring community-based oral health programs. Existing frameworks, such as Costa Rica’s National Oral Health Program, could be enhanced by incorporating anatomical risk-based strategies, including ICDAS-guided risk identification, targeted sealant application, and early parental education. International and regional models—such as Brazil’s integration of oral health into primary care and multicenter initiatives across Latin America—serve as valuable benchmarks for improving community-based pediatric dental care and enhancing children's oral health–related quality of life [20, 38].

One of the key strengths of this study is the use of the ICDAS system, which enables detailed classification of lesion severity, ranging from noncavitated to extensively cavitated stages. The inclusion of all primary teeth in a nationally representative sample of children attending public early childhood centers enhances the generalizability of the findings to vulnerable pediatric populations in Costa Rica.

This study has several limitations. As a cross-sectional analysis, it does not allow for inferences about causality or lesion progression over time. The absence of radiographic data may have led to the underestimation of early interproximal lesions. In addition, fluoride exposure, dietary habits, and dental service utilization were not assessed, despite their potential influence on caries development and severity. Although examiner calibration was conducted and inter- and intra-examiner reliability were high, some degree of diagnostic variability cannot be entirely excluded. Finally, although the response rate was high, selection bias is possible if children absent on the day of examination differed systematically from those included.

Future studies should adopt longitudinal cohort designs to better understand the natural history of caries and the effectiveness of targeted interventions. Incorporating data on dietary habits, oral hygiene behaviors, and parental socioeconomic indicators would offer deeper insights into the social determinants of anatomical lesion patterns. Overall, anatomical mapping of dental caries by tooth and surface provides a powerful framework for guiding clinical decision-making and shaping public health strategies. Integrating surface-level ICDAS surveillance into national programs could enhance early detection, prioritize high-risk sites, and enable cost-effective, risk-based interventions for vulnerable preschool populations.

## Conclusions

The findings of this study demonstrated that second primary molars are the most frequently affected teeth among Costa Rican children attending CEN-CINAI centers, whereas upper central incisors, when affected, exhibit the greatest lesion severity. At the surface level, occlusal surfaces were the most commonly affected, followed by buccal and mesial surfaces. This progression pattern highlights the importance of early detection in anatomically challenging areas. These results reinforce the need for preventive strategies tailored to anatomical risk and expected lesion severity by tooth and surface type.

The clinical and public health implications of this analysis are clear: intensified preventive efforts are needed for primary molars, and early detection is essential for preventing the progression of carious lesions in anterior teeth. In addition, oral health programs in Costa Rica should incorporate targeted actions focused on the most vulnerable teeth and surfaces, complementing universal fluoride and sealant strategies with personalized, risk-based approaches. These findings offer a solid foundation for guiding both clinical practice and public policy development aimed at ensuring comprehensive protection of children's oral health.

## Data Availability

The datasets generated and/or analysed during the current study are available from the corresponding author on reasonable request.

## Abbreviations

ECC: Early childhood caries
ICDAS: International Caries Detection and Assessment System
Dmfs: Decayed, missing, and filled surfaces
CEN-CINAI: Centros de Educación y Nutrición y Centros Infantiles de Atención Integral (Education and Nutrition Centers and Comprehensive Child Care Centers)
PPS: Probability Proportional to Size
SPSS: Statistical Package for the Social Sciences
CI: Confidence Interval
